# Validation of the AOSpine-DGOU Osteoporotic Fracture Classification – Effect of Surgical Experience, Surgical Specialty, Work-Setting and Trauma Center Level on Reliability and Reproducibility

**DOI:** 10.1177/21925682251331945

**Published:** 2025-03-24

**Authors:** Julian Scherer, Sebastian Frederick Bigdon, Gaston Camino-Willhuber, Ulrich Spiegl, Andrei Fernandes Joaquim, Harvinder Singh Chhabra, Marcel Dvorak, Gregory Schroeder, Mohammad El-Sharkawi, Richard Bransford, Lorin Michael Benneker, Klaus John Schnake, on behalf of the AO Spine-DGOU International Validation Group

**Affiliations:** 1 Orthopaedic Research Unit, University of Cape Town, Cape Town, South Africa; 2 Department of Traumatology, 27217 University Hospital of Zurich, Zürich, Switzerland; 3 Department for Orthopaedics and Traumatology, Inselspital, 27252 University Hospital Bern, Bern, Switzerland; 4 Department for Spine Surgery, Sonnenhof Spital, University of Bern, Bern, Switzerland; 5 Policina Gipuzkoa, San Sebastian, Spain; 6 Klinik für Unfallchirurgie und Orthopädie, Klinik München Harlaching, München, Germany; 7 Neurosurgery Division, Department of Neurology, State University of Campinas, Campinas-Sao Paulo, Brazil; 8 309539 Sri Balaji Action Medical Institute, New Delhi, India; 9 Combined Neurosurgical and Orthopedic Spine Program, 8167 Vancouver General Hospital, University of British Columbia, Vancouver, BC, Canada; 10 Department of Orthopaedic Surgery, Rothman Institute, Thomas Jefferson University Hospital, Philadelphia, PA, USA; 11 Department of Orthopaedic and Trauma Surgery, Faculty of Medicine, 68796 Assiut University, Assiut, Egypt; 12 Department of Orthopaedics and Sports Medicine, 21618 Harborview Medical Center, University of Washington, Seattle, WA, USA; 13 Center for Spinal and Scoliosis Surgery, 232691 Malteser Waldkrankenhaus St. Marien Erlangen, Erlangen, Germany; 14 Department of Orthopedics and Traumatology, Paracelsus Private Medical University Nuremberg, Nuremberg, Germany

**Keywords:** osteoporosis, spinal fracture, classification, validation study, osteoporotic vertebral compression fractures

## Abstract

**Study Design:**

Cross-sectional survey.

**Objectives:**

A cornerstone of classification systems is good reliability amongst different groups of classification users. Thus, the aim of this international validation study was to assess the reliability of the new AO Spine DGOU Osteoporotic Fracture Classification (OF classification) stratified by surgical specialty, work-setting, work-experience, and trauma center level.

**Methods:**

320 spine surgeons were asked to rate 27 cases according to the OF classification at 2 time points, 4 weeks apart (assessment 1 and 2) in this online-webinar based validation process. The kappa statistic (κ) was calculated to assess the inter-observer reliability and the intra-rater reproducibility.

**Results:**

A total of 7798 (90.3%) ratings were recorded in assessment 1 and 6621 (76.6%) ratings in assessment 2. Global inter-rater reliability was moderate in both assessments (κ = 0.57; κ = 0.58). Participants with a work-experience of >20 years showed the highest inter-rater agreement in both assessments globally (κ = 0.65; κ = 0.67). Participants from a level-1 trauma center showed the highest agreement (κ = 0.58), whereas participants working at a tertiary trauma center showed higher grade of agreement in the second assessment (κ = 0.66). Participants working in academia showed the highest agreement in assessment 2 (κ = 0.6). Surgeons with academic background and surgeons employed by a hospital showed substantial intra-rater agreement in the second assessment.

**Conclusions:**

The AO Spine-DGOU Osteoporotic Fracture Classification showed moderate to substantial inter-rater agreement as well as intra-rater reproducibility regardless of work-setting, surgical experience, level of trauma center and surgical specialty.

## Background

Osteoporosis represents an increasing public health concern worldwide in the elderly population.^1^ Due to the potential morbidity and mortality, osteoporotic vertebral fractures (OVFs) are of increasing concern as they represent the most common osteoporotic fractures due to low bone density and fragility.^2,3^ With the rising incidence and prevalence of OVFs, it is crucial to implement evidence-based treatment algorithms in order to establish standardized care strategies for these patients.^4,5^ These treatment algorithms are mainly based on fracture classifications to determine fracture morphology precisely, which also facilitates uniform communication between caregivers and guide in determining optimal treatment strategies. AO (Arbeitsgemeinschaft für Osteosynthesefragen) Spine developed and introduced a new classification system for thoracolumbar fractures in 2013, which was quickly accepted and validated with high reproducibility internationally.^6,7^ However, this classification was not developed specifically for patients with osteoporotic changes. A distinct differentiation between high-energy vertebral fractures and OVFs is crucial, since OVFs are being managed differently due to their unique prognosis and characteristics.^8^ The Spine Section of the German Society for Orthopaedics and Trauma (DGOU), who have formed the working group “Osteoporotic Fractures”, proposed a new classification for osteoporotic thoraco-lumbar spine fractures.^3^ Further, the final AO Spine-DGOU Osteoporotic Fracture (OF) classification system was developed after several consensus meetings with members of the AO Spine Knowledge Forum Trauma. This classification (OF) consists of 5 groups: OF 1, no deformation, only bone edema assessed with MRI, OF 2, deformation of 1 endplate with no or minor involvement of posterior wall, OF 3, deformation of 1 endplate with substantial involvement of posterior wall, OF 4, deformation of both endplates with or without involvement of posterior wall, and OF 5, fracture with anterior or posterior tension band failure. (Appendix 1).

Based on this classification system, treatment recommendations were proposed by Blattert et al and subsequently validated in a large multi-center study from Germany. The study showed high accordance in treatment strategies of OVFs with favorable outcomes with the proposed treatment recommendations.^8,9^ A cornerstone of classification systems is good reliability amongst different groups of classification users (eg, different levels of surgical experience).^10^ Studies on the reliability of subaxial and thoracolumbar AO Spine classifications have shown that the spine surgeon`s level of experience and subspecialty did not have substantial influence on the classification and intra-observer reliability of these classifications.^11,12^ However, data on the reliability amongst different surgeons` levels of experience for the AO Spine-DGOU OF classification is lacking. Thus, the aim of this international validation was to assess the reliability of the AO Spine-DGOU OF classification amongst spine surgeons with different levels of experience and subspecialties as well as to assess the effect of the level of trauma center spine surgeons are working at on the reliability of this classification.

## Material and Methods

### Development of Classification System

The methodology behind the creation of the AO Spine-DGOU OF classification has been described in detail in the publication of the Spine Section of the German Society for Orthopaedics and Trauma (DGOU).^3^

### Validation Process

The present international validation followed strictly the previously published methodology and recommendation of Lambrechts et al.^13^ In total, 320 spine surgeons participated in this online-webinar based validation. All participants were invited in advance to watch a video on the correct application and classification. Additionally, the official poster of the AO Spine-DGOU OF classification for illustration was available. Before the start of the validation, all participants were given the opportunity to ask questions to the instructor. After a short introduction, the participants were given the opportunity to practice the handling in the context of training cases. Subsequently, the 27 cases were presented by means of a key-image and subsequently by means of CT and (if available or relevant) MRI in sagittal, coronal and axial slices. The participants were then able to give their rating for the respective case in RedCap (REDCap Software – Version 6.5.2 - © 2023 Vanderbilt University).

The second rating took place with rearranged cases 4 weeks after the initial assessment to avoid recall bias.

### Gold-Standard-Committee

Members of the Osteoporotic Fracture working group of the DGOU provided the gold-standard classification for each injury on radiological imaging. Prior to evaluation of the injuries by the study participants, the Gold Standard Committee reached unanimous agreement on the classification of each presented case.

### Statistical Analysis

The kappa statistic (κ) was calculated to assess the reliability of the classification system among different observers (inter-rater agreement) and the reproducibility for the same observer on separate occasions (intra-rater reproducibility).^14^ The Fleiss kappa statistic measures the agreement of multiple raters who rate multiple subjects, with the rating based on multiple categories. The coefficients were interpreted utilizing the Landis and Koch grading system, which defines κ ≤ 0.2 as slight reliability (agreement/reproducibility), 0.2 < κ ≤ 0.4 as fair reliability, 0.4 < κ ≤ 0.6 as moderate reliability, 0.6 < κ ≤ 0.8 as substantial reliability, and κ > 0.8 as excellent reliability.^15^ Inter-rater and intra-rater agreement was calculated for the different classification groups (OF 1-5) and overall, for the classification itself. Furthermore, agreement with the gold-standard (accuracy) was calculated overall and for each OF-subtype. Results were stratified by the raters` specialty, work-experience in years (subgroups: <5 years; 5-10 years; 11-20 years; >20 years), work-setting and level of trauma center. Differences between the groups were calculated using the chi-square test. Statistical significance was set at *P* < 0.05.

## Results

### Participants and Ratings

A total of 320 spine surgeons participated in this validation of 27 cases at 2 occasions 4 weeks apart with identical cases in different order. The fractures were classified after gold- standard evaluation prior this validation. (Table 1).

**Table 1.** Distribution of Presented Cases According to the AO Spine-DGOU Osteoporotic Fracture Classification.

Classification	N	%
OF 1	2	7.4
OF 2	7	25.9
OF 3	4	14.8
OF 4	9	33.3
OF 5	5	18.5
Total	27	100

Out of 17280 possible ratings (8640 ratings in each validation time point), 7798 (90.3%) ratings were recorded in the first validation round and 6621 (76.6%) ratings were recorded 4 weeks later.

Most of the Participants Reported a Work Experience of 5 to 10 years (n = 97, 30.3%).

Orthopaedic spine surgery was the most frequently reported subspecialty amongst the participants (n = 182, 56.9%) and most of the study participants were hospital employed (n = 142, 44.4%).

Most of the participants (n = 198, 61.9%) stated that they were working at a level-1 trauma center (highest level of injury care). (Table 2).

**Table 2.** Distribution of Participants Stratified by Time in Practice, Subspecialty, Trauma Center Level and Work-Setting.

Time in Practice (years)	N	%
<5	75	23.4
5 – 10	97	30.3
11 – 20	93	29.1
>20	55	17.2
Total	320	100
**Subspecialty**		
Neurosurgery	111	34.7
Orthopaedic spine surgery	182	56.9
Other	27	8.4
Total	320	100
**Work-setting**		
Academic	136	42.5
Hospital employed	142	44.4
Private practice	42	13.1
Total	320	100
**Trauma center level**		
I	198	61.9
II	72	22.5
III	23	7.2
IV	13	4.1
No trauma	14	4.4
Total	320	100

### Agreement With Gold-Standard

**Figure 1.**
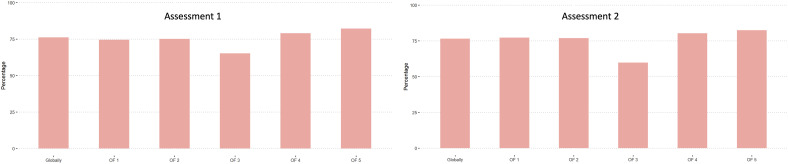
Agreement with gold-standard amongst all participants stratified by OF-classification.

Amongst all participants (unstratified), the evaluation showed relatively high agreement with the gold-standard in both, first and second assessment. (Figure 1).

Regarding time in practice, the highest global agreement with the gold-standard was achieved in the group practicing more than 20 years in both, assessment 1 and assessment 2. OF 3 fractures showed the lowest agreement with the gold-standard amongst all time in practice groups.

Overall, the highest agreement with the gold-standard was achieved by neurosurgeons in assessment 1 (76.9%) and by orthopaedic spine surgeons (77.5%) in assessment 2. The least agreement with the gold-standard amongst the different subspecialties was found in OF 3 fractures. Generally, the agreement with gold-standard increased from the first to the second assessment for neurosurgeons and orthopaedic spine surgeons but decreased for the “other” group.

Surgeons from level-1 trauma centers achieved the highest agreement globally in assessment 1 (76.6%), whereas level-3 centers achieved the best agreement with the gold-standard in the second assessment (81.5%). Also in this sub analysis, fractures classified as OF 3 were rated the poorest.

Hospital employed participants achieved the best agreement with the gold-standard globally in the first assessment (77.3%), whereas surgeons from an academic work environment had the best agreement with the gold-standard in the second assessment (76.9%).

All 4 subgroups showed a decrease in OF 3 classified fracture agreement with the gold-standard from the first to the second assessment. OF 4 and OF 5 were generally rated the best in comparison to the gold-standard amongst all subgroups. Detailed statistical analysis can be found in Table 3.

**Table 3.** Agreement With Gold-Standard (%) Stratified by Time in Practice, Subspecialty, Trauma Center Level and Work-Setting; AS1 = Assessment 1; AS2 = Assessment 2; NS = Neurosurgery; OSS = Orthopaedic Spine Surgeon; Level IV and No-Trauma Centers Have Been Merged for Statistical Reasons; Comparison of Subspecialty was Performed for Orthopaedic Surgeons Versus Neurosurgeons; *Indicates Statistical Significance (*P* < 0.05).

Classification	<5y		5-10y		11-20y		>20y		*P*-value	
	*AS1*	*AS2*	*AS1*	*AS2*	*AS1*	*AS2*	*AS1*	*AS2*	*AS1*	*AS2*
Globally	69.2	69.7	78.1	77.4	76.7	77.4	81.0	81.7	<0.0001*	<0.0001*
OF 1	70.8	71.3	74.5	75.8	75.4	81.3	78.3	79.5	0.633	0.274
OF 2	65.4	67.5	76.3	79.1	77.1	76.7	82.5	85.0	<0.0001*	<0.0001*
OF 3	58.7	50.0	65.9	60.8	64.2	61.5	74.1	67.5	0.008*	0.005*
OF 4	74.1	77.9	80.4	79.7	79.7	79.7	81.9	85.2	0.008*	0.067
OF 5	73.7	73.5	87.8	84.9	81.4	85.5	84.0	83.1	<0.0001*	0.0004*

	NS		OSS		Other		*P*-value	
Globally	*76.9*	*77.1*	*76.6*	*77.5*	*69.5*	*64.6*	0.8419	0.7299
OF 1	*71.0*	*74.3*	*76.9*	*80.1*	*72.7*	*68.8*	0.1586	0.1810
OF 2	*78.2*	*79.3*	*74.6*	*76.8*	*65.0*	*64.0*	*0.0869*	0.2615
OF 3	*65.8*	*60.3*	*65.7*	*61.2*	*57.8*	*45.5*	*1.0000*	0.8359
OF 4	*78.4*	*80.5*	*79.8*	*81.3*	*75.1*	*69.4*	*0.4447*	0.6754
OF 5	*83.5*	*82.5*	*82.4*	*83.3*	*73.9*	*70.7*	*0.6727*	0.6006

	Level 1		Level 2		Level 3		Level 4/no trauma		*P*-value	
Globally	*76.6*	*77.5*	*75.2*	*72.2*	*76.2*	*81.5*	*75.7*	*76.3*	*0.674*	<0.0001*
OF 1	*73.3*	*77.3*	*78.9*	*77.0*	*81.6*	*72.2*	*65.9*	*81.6*	*0.230*	*0.819*
OF 2	*75.9*	*78.1*	*75.6*	*70.6*	*69.2*	*86.3*	*71.9*	*76.7*	*0.286*	0.001*
OF 3	*63.6*	*59.1*	*66.7*	*57.5*	*67.5*	*66.7*	*71.4*	*66.2*	*0.445*	*0.334*
OF 4	*70.6*	*81.7*	*76.9*	*76.1*	*80.1*	*83.1*	*78.9*	*77.8*	0.561	0.034*
OF 5	*83.8*	*84.1*	*76.6*	*77.4*	*84.0*	*87.6*	*82.9*	*79.2*	0.031*	0.035*

	Academic		Hospital employed		Private practice		*P*-value	
Globally	75.0	76.9	77.3	76.2	76.5	75.9	0.085	0.756
OF 1	74.4	76.4	73.7	77.0	77.8	80.6	0.782	0.779
OF 2	73.4	77.5	77.0	75.8	74.1	78.4	0.205	0.639
OF 3	62.7	59.8	68.0	59.3	63.0	61.6	0.181	0.896
OF 4	79.1	80.9	79.5	80.7	76.5	75.9	0.498	0.153
OF 5	79.7	82.8	82.3	82.2	90.1	81.9	0.007*	0.948

### Inter-Rater Agreement

Amongst all participants, the global inter-rater reliability was moderate in both, first and second assessment (κ = 0.57; κ = 0.58). The best inter-rater reliability globally was achieved for fracture subtypes OF 4 in both assessments (κ = 0.68; κ = 0.70), whilst the worst reliability was found in OF 3 fractures in both assessments (κ = 0.44; κ = 0.42).

The subgroup with a work-experience of more than 20 years showed the highest inter-rater agreement amongst all participations in both assessments globally (κ = 0.65; κ = 0.67). In most of the fracture types, there was a general improvement between the assessments amongst all work experience groups, however, only the more experienced surgeons showed improvement in OF 3 fracture types.

Neurosurgeons had the best global inter-rater agreement in the first assessment (κ = 0.59) whereas orthopaedic spine surgeons showed a higher agreement in assessment 2 (κ = 0.60). The “other” group showed general decrease in reliability amongst all fracture types in the second assessment compared to the first, whereas neurosurgeons and orthopaedic spine surgeons showed general improvement.

In the first assessment, participants working at a level-I trauma center showed the highest agreement (κ = 0.58), whereas participants working at a tertiary trauma center showed higher grade of agreement in the second assessment (κ = 0.66). There was a general improvement amongst trauma levels except for OF 3 fracture types amongst level-I and -II centers and level-III as well as level-IV/no trauma centers showed the highest increase of agreement compared to the first assessment.

Participants working in a private practice showed the highest agreement in the first assessment (κ = 0.59), while participants working in academia showed the highest agreement in the second assessment (κ = 0.6). Surgeons from private practices showed the highest decrease in agreement between the assessments compared to surgeons with academic background and surgeons employed by a hospital.

For all analyzed subgroups, OF 3 showed the lowest reliability in both assessments, whereas OF 4 fractures showed the highest reliability. Detailed statistical analysis can be found in Table 4.

**Table 4.** Inter-rater Reproducibility (κ) Stratified by Time in Practice, Subspecialty, Trauma Center Level and Work-Setting; AS1 = Assessment 1; AS2 = Assessment 2; NS = Neurosurgery; OSS = Orthopaedic Spine Surgeon; Level IV and No-Trauma Centers Have Been Merged for Statistical Reasons.

Classification	<5y		5-10y		11-20y		>20y	
	*AS1*	*AS2*	*AS1*	*AS2*	*AS1*	*AS2*	*AS1*	*AS2*
Globally	0.45	0.47	0.61	0.61	0.58	0.60	0.65	0.67
OF 1	0.44	0.46	0.57	0.62	0.56	0.61	0.67	0.65
OF 2	0.44	0.47	0.56	0.58	0.54	0.56	0.64	0.66
OF 3	0.33	0.26	0.47	0.46	0.43	0.44	0.58	0.59
OF 4	0.56	0.61	0.71	0.72	0.71	0.71	0.73	0.78
OF 5	0.43	0.47	0.65	0.62	0.57	0.62	0.62	0.63

	NS		OSS		Other	
Globally	0.59	0.60	0.57	0.60	0.45	0.37
OF 1	0.56	0.58	0.56	0.61	0.46	0.37
OF 2	0.58	0.58	0.53	0.56	0.41	0.40
OF 3	0.45	0.46	0.45	0.44	0.32	0.16
OF 4	0.69	0.70	0.69	0.73	0.57	0.51
OF 5	0.59	0.61	0.57	0.61	0.43	0.33

	Level 1		Level 2		Level 3		Level 4/no trauma	
Globally	0.58	0.61	0.55	0.50	0.57	0.66	0.53	0.56
OF 1	0.54	0.61	0.60	0.51	0.59	0.63	0.43	0.56
OF 2	0.56	0.59	0.53	0.47	0.49	0.69	0.47	0.50
OF 3	0.45	0.44	0.40	0.34	0.49	0.52	0.41	0.46
OF 4	0.69	0.72	0.65	0.63	0.68	0.74	0.66	0.71
OF 5	0.58	0.61	0.55	0.52	0.59	0.65	0.58	0.53

	Academic		Hospital employed		Private practice	
Globally	0.55	0.60	0.58	0.57	0.59	0.56
OF 1	0.56	0.59	0.53	0.57	0.58	0.59
OF 2	0.50	0.59	0.58	0.54	0.55	0.54
OF 3	0.41	0.43	0.47	0.43	0.44	0.38
OF 4	0.68	0.71	0.68	0.69	0.69	0.70
OF 5	0.55	0.61	0.57	0.58	0.63	0.55

### Intra-Rater Reproducibility

**Figure 2.**
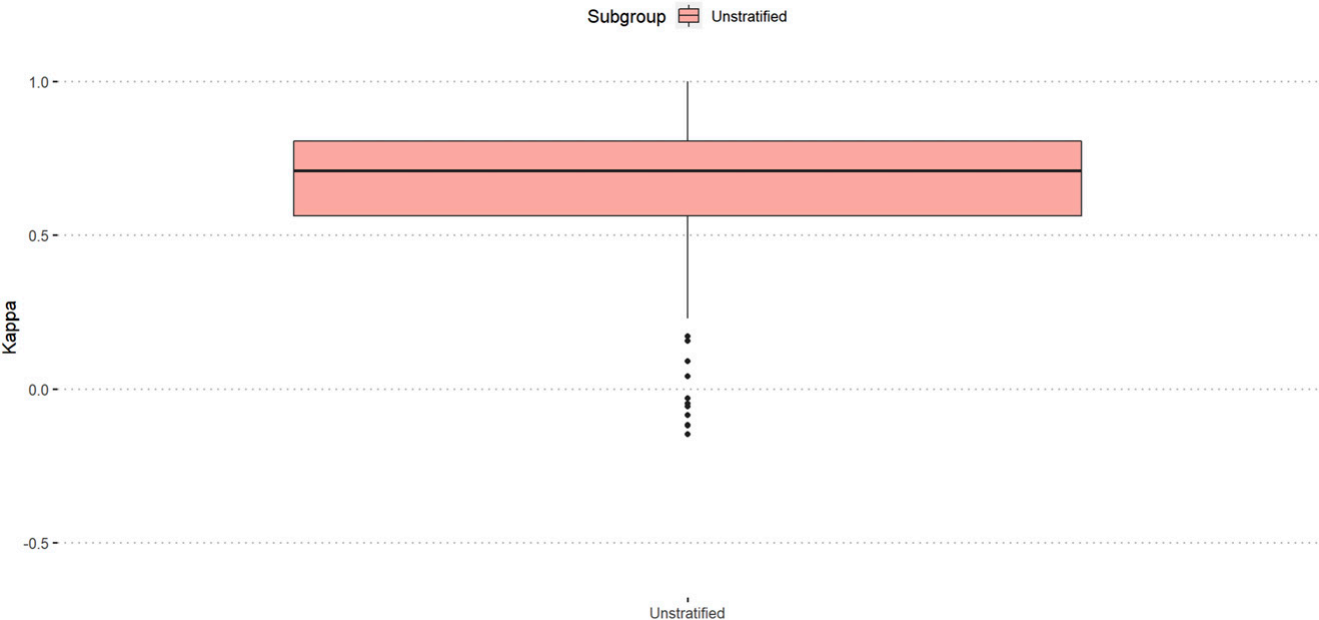
Intra-rater reliability amongst all participants, unstratified.

Amongst all participants (n = 234), the median intra-rater reliability was substantial (κ = 071). (Figure 2).

**Figure 3.**
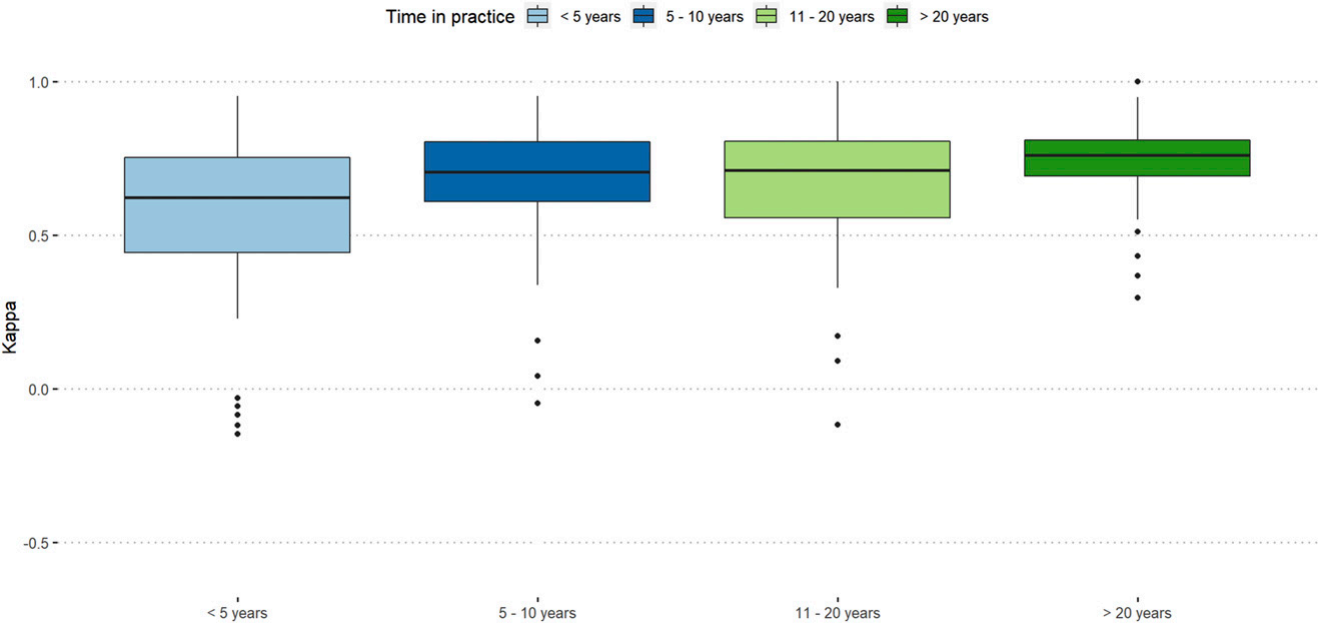
Intra-rater reliability stratified by work-experience.

**Figure 4.**
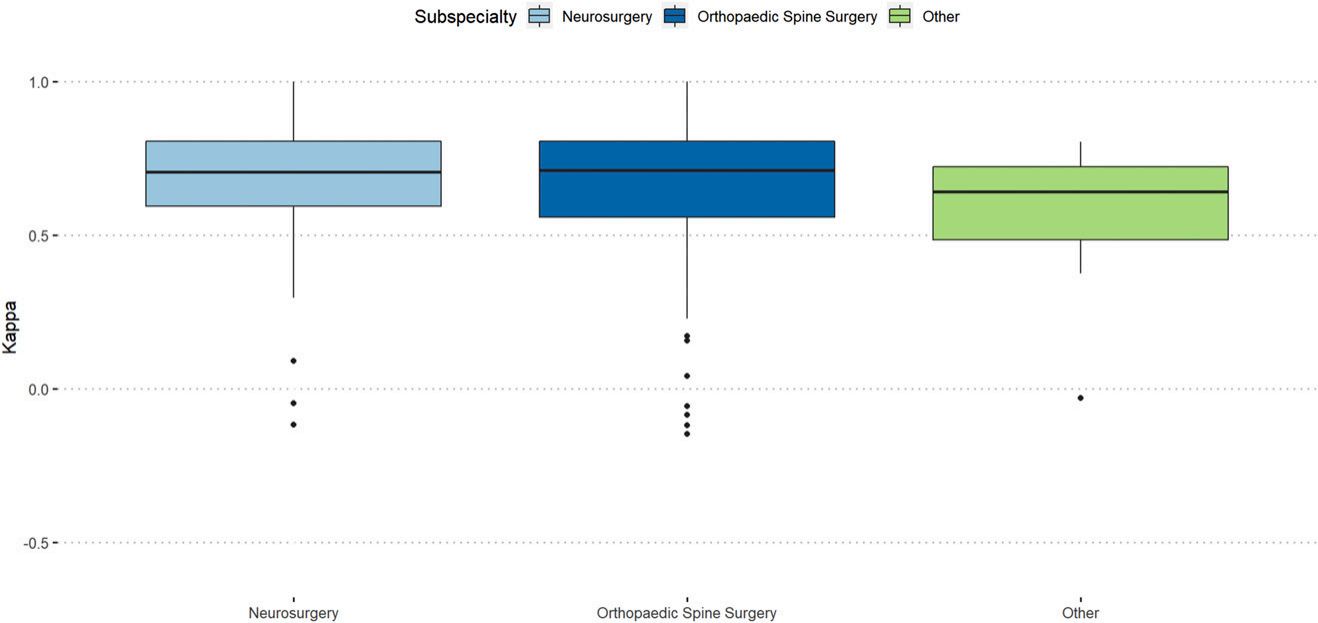
Intra-rater reliability stratified by surgical specialty.

Participants with more than 20 years of work-experience showed almost excellent reproducibility (κ = 0.76) and orthopaedic spine surgeons showed slightly better intra-rater reproducibility than neurosurgeons (κ = 0.71 vs κ = 0.7). (Figures 3 and 4).

**Figure 5.**
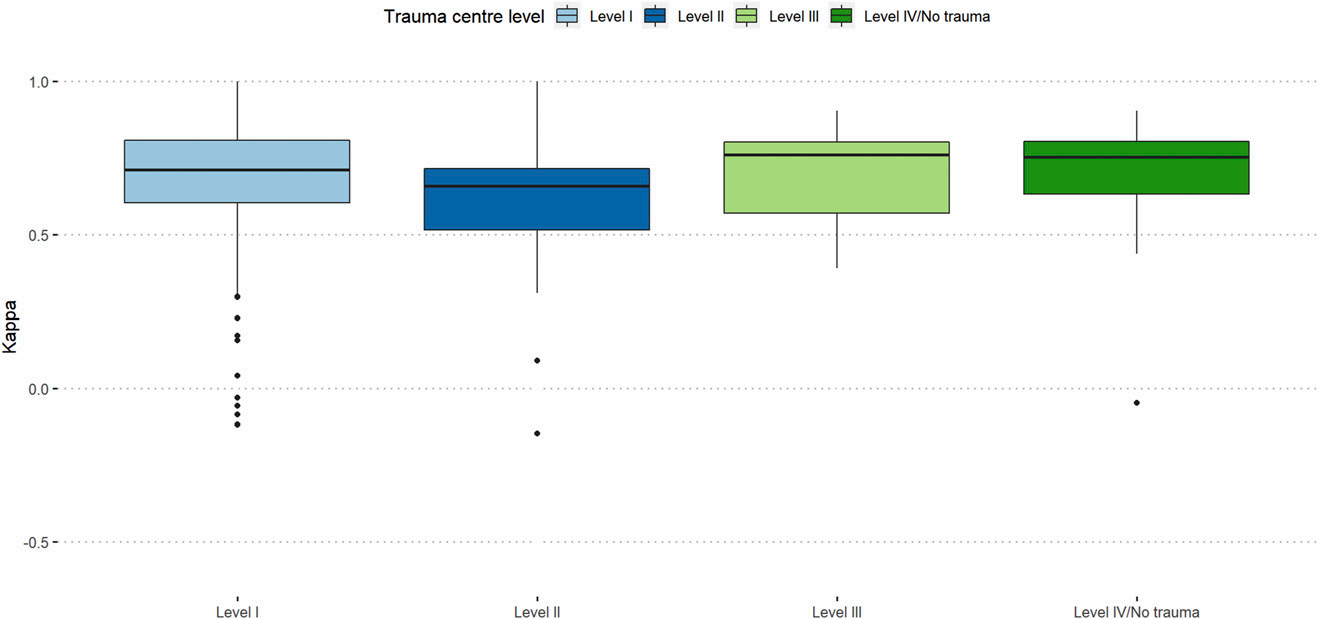
Intra-rater reliability stratified by trauma center level.

**Figure 6.**
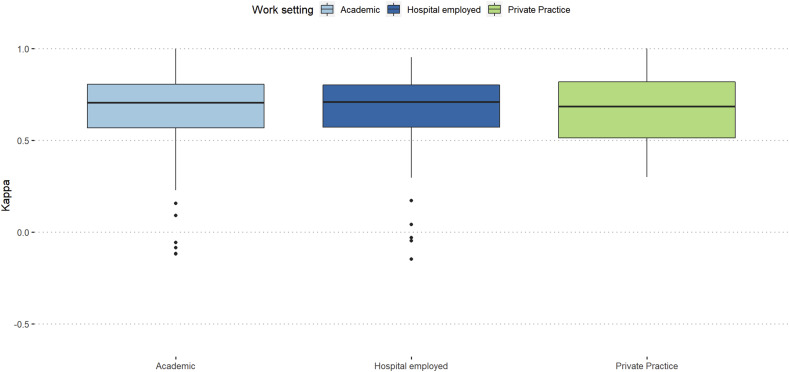
Intra-rater reliability stratified work-setting.

Participants working at level-3 trauma centers showed the best intra-rater reproducibility (κ = 0.76), and participants from academic background and hospital employed surgeons showed similar intra-rater reproducibility (κ = 0.71). (Figures 5 and 6).

## Discussion

The aim of this international validation of the AO Spine-DGOU OF classification was to assess the effect of surgeon`s subspecialty, work-experience, work-setting, and level of trauma center on the reliability and reproducibility of this classification. We assessed several interesting findings.

### Comparison With the Gold-Standard

In general, we found a relatively good global agreement with the gold-standard, which was slightly inferior compared to evaluations of other AO Spine classifications.^16,17^ This phenomenon might be explained due to the relative novelty of the OF-classification. OF-3 fractures were rated the poorest and OF-4 fractures were rated best amongst the participating raters. This might be due to the borderline-nature of OF-3 fractures, which can be either over-interpreted as OF-4 or under-interpreted as OF-2. This has been shown in previous studies assessing only fair agreement in OF-3 fractures.^18,19^ However, this observation is crucial for refining and enhancing classification systems in future studies and specifically re-defining the OF-3 fracture. More experienced surgeons were significantly more in agreement with the gold-standard of the OF-classification in this evaluation compared to less experienced surgeons, which is similar to the evaluation of the AO Spine Upper Cervical Spine Injury Classification System and the AO Spine sacral classification system but contrary to the evaluation of the AO Spine Thoracolumbar Spine Injury Classification System.^11,12,20^ The better agreement with the gold-standard in more experienced surgeons might be due to better radiological knowledge which is needed for the OF-classification and favors work-experience. This finding suggests that the implementation of early training of this classification might have a positive effect on rating success. Raters from level-3 trauma centers had a significant learning curve in between the 2 assessments and showed similar agreement with the gold-standard as level-1 trauma centers in the first assessment and even better agreement compared to level-1 centers in the second assessment. The better agreement in raters from level-1 centers might be explained due to general higher case load, however, since the OF classification is imaging based, the better agreement in level-3 centers might be due to the scarcity of available radiologists and that spine surgeons from these centers might be better trained in analyzing acquired imaging without a radiologist at site. A previous study on the reliability of the upper cervical spine injury classification has shown significantly higher agreement with the classification gold-standard in level-1 and level-2/3 trauma centers compared to level-4/no trauma centers, which we were not able to assess in this evaluation.^21^ Overall, there was no significant difference regarding work-setting in this evaluation on the agreement with gold-standard, which is inconsistent with evaluation on other AO Spine classifications.^21^ Further, we did not assess significant differences between the spine subspecialties regarding overall agreement with the gold-standard, which is consistent with the evaluation on the AO Spine upper cervical spine classification and the AO Spine sacral classification.^12,20^

### Inter-Rater Agreement

Amongst all participants, the global inter-rater reliability was moderate in both assessment (first:κ = 0.57; second:κ = 0.58). The same could be shown within 2 previous studies assessing the inter-rater agreement of this classification amongst 6 surgeons in each study.^18,22^ Furthermore, we found that our findings are comparable to the previous international validation study of the AO Spine thoracolumbar injury classification system (κ = 0.56).^23^

Amongst all participants and subgroups, fractures classified as OF-3 according to the OF classification showed moderate inter-rater agreement in both assessment (κ = 0.44; κ = 0.42), whereas OF-4 fractures produced substantial agreement (κ = 0.68; κ = 0.70) which is a similar finding as the comparison with the gold-standard. Globally, participants with less than 5 years of work-experience showed moderate inter-rater agreement in both assessments (κ = 0.45; κ = 0.47), whereas all other participants showed substantial inter-rater agreement. The best performance was observed within in the group of participants with more than 20 years experience. These findings are similar to an assessment of the AO Spine upper cervical spine classification, however, in this previous assessment, participants with more than 20 years of work experience were not able to reach the threshold of substantial inter-rater agreement at the second assessment.^12^ Furthermore, our assessment showed inferior agreements compared to the AO Spine sacral classification, where throughout all experience groups, an excellent agreement was achieved.^20^ In an assessment of the AO Spine thoracolumbar injury classification system, participants with more than 20 years of experience showed the poorest inter-rater agreement compared to participants with least experience, which is contrary to our findings.^11^ Both, neurosurgeons and orthopaedic spine surgeons showed substantial inter-rater agreement in the second assessment (κ = 0.60), whereas other surgeons showed only fair agreement (κ = 0.37), which is a similar finding to the assessment of the AO Spine upper cervical injury classification.^12^ This seems logical, since other specialties such as general surgeons are less frequently in contact with spinal injuries and therefore are in less need of classifying these injuries. However, proper training of non-spine specialties might facilitate a common language between several specialties. Interestingly, only level-1 and level-3 trauma centers showed substantial inter-rater agreement (κ = 0.61 and κ = 0.66) in the second assessment, which is partly in line with an assessment of the AO Spine upper cervical injury classification (κ = 0.66 and κ = 0.58). ^21^ It must be noted that this previous assessment analyzed level-2 and -3 trauma centers jointly and therefore, this data can be misleading. The reason for the better inter-rater agreement in the level-3 center group might be the same as described for the comparison to gold-standard. In this assessment, participants from an academic background showed the best inter-rater agreement in the second assessment (κ = 0.60), which is in keeping with the assessment of the AO Spine upper cervical injury classification.^21^ These findings might be explained by the increased contact with scientific research and therefore classification of participants with an academic background as well as increased contact with acute trauma patients and higher patient load in general.

### Intra-Rater Reliability

We assessed a substantial overall intra-rater agreement amongst all participants (κ = 0.71), which reflects the practicality of the OF classification.

Participants with a surgical experience of more than 20 years showed almost excellent reliability (κ = 0.76), which is contrary to the validation of the AO Spine upper cervical injury classification system, where the best intra-rater reliability was achieved by participants with surgical experience of less than 5 years, and also contrary to the validation of the AO Spine thoracolumbar injury classification system, where participants with an experience of 11-20 years achieved the highest reliability.^11,12^

Similar to the comparison to gold-standard and inter-rater agreement in this study, participants from level-3 trauma centers showed the highest intra-rater reliability (κ = 0.76) which is contrary to other AO Spine classification systems.^21^

In our assessment, participants with academic background and participants who were hospital employed, showed the same reliability (κ = 0.71), which is a similar findings compared to the validation of the AO Spine upper cervical injury classification.^21^

Orthopaedic spine surgeons showed a slightly better intra-rater reliability (κ = 0.71) than neurosurgeons (κ = 0.70) and others (κ = 0.64), which is in keeping with other previously validated AO Spine classifications.^12,17^

### Limitations

This study has certain limitations. Firstly, there might be a varying degree of familiarity with the OF-classification amongst the raters which results in a classification knowledge-inequality and could result in a potential bias. Furthermore, the sole use of key-images (in some cases) or vice versa might have an impact on the rater`s response. It is likely that some of the reviewers would change their response while assessing a complete imaging file rather than solely key images. Another potential limitation is the inequal frequency of different fracture subtypes and hence, the data on rare subtypes should be interpreted with caution. Additionality, some of the presented images showed multiple fractures which might have caused confusion regarding the fracture to rate, decreasing the reliability rate. However, it was mentioned beforehand, that the most apparent fracture was to rate in order to reduce bias. Furthermore, the members of the AO Spine Knowledge Forum Trauma might have varying degrees of knowledge on interpreting different imaging modalities and familiarity with the presented classification since not all members were involved in the development process, which could result in an inhomogeneous group of raters and could have a negative effect on the inter-rater agreement. Standardized training on imaging and of the OF-classification could limit these potential biases. Further international validation with a larger, more diverse group of surgeons is needed.

## Conclusions

Longer work experience was associated with higher inter-rater agreement and intra-rater reliability. This finding may suggest implementing training of the assessed classification in the early stages of the surgical career. Surgical subspecialties other than orthopaedic spine surgeons and neurosurgeons showed inferior ratings, which highlights the necessity of teaching this new classification amongst other none-spine specialties. Level-1 and Level-3 trauma centers showed good results in both, inter-rater agreement, and intra-rater reproducibility. Overall, the AO Spine-DGOU Osteoporotic Fracture Classification showed moderate to substantial inter-rater agreement as well as intra-rater reproducibility regardless of work-setting, surgical experience, level of trauma center and surgical specialty.

## Supplemental Material

Supplemental Material - Validation of the AOSpine-DGOU Osteoporotic Fracture Classification – Effect of Surgical Experience, Surgical Specialty, Work-Setting and Trauma Center Level on Reliability and Reproducibility Supplemental Material for Validation of the AOSpine-DGOU Osteoporotic Fracture Classification – Effect of Surgical Experience, Surgical Specialty, Work-Setting and Trauma Center Level on Reliability and Reproducibility by Julian Scherer, Sebastian Frederick Bigdon, Gaston Camino-Willhuber, Ulrich Spiegl, Andrei Fernandes Joaquim, Harvinder Singh Chhabra, Marcel Dvorak, Gregory Schroeder, Mohammad El-Sharkawi, Richard Bransford, Lorin Michael Benneker, Klaus John Schnake, and on behalf of the AO Spine-DGOU international validation Group in Global Spine Journal

## Data Availability

Data is available upon reasonable request towards the corresponding author.*
